# False Elevation of Troponin Levels in a Patient With Chest Pain and Significant Psychoemotional Stress

**DOI:** 10.7759/cureus.47223

**Published:** 2023-10-17

**Authors:** Ali Elkhedr, Sabri Elkhidir

**Affiliations:** 1 Internal Medicine, Marshfield Medical Center, Marshfield, USA

**Keywords:** false-positive, acute myocardial infarction, diagnostic challenge, cardiac troponin, false positive troponin

## Abstract

We present a case of a 63-year-old woman who experienced significant psychoemotional stress and was seen in the emergency department for an evaluation of chest pain. She was sent home after reassurance. Subsequent troponin levels came back above the normal range. The patient was called back to the hospital for further assessment, including a potential workup for the exclusion of acute coronary syndrome, given the existing risk factors, symptoms, and elevated cardiac enzymes. The patient has risk factors for atherosclerosis, including hypercholesterolemia, hypertension, and age. She has recently gone through hard personal, emotional, and psychosocial stress. She presented with atypical chest pain, subjective sense of breathlessness, and anxiety. She was reassured, had blood tests ordered, and was discharged after relative improvement. Later that day, her blood test results, which were taken earlier, showed elevated troponin levels. Therefore, she was asked to come back for a cardiology assessment and underwent coronary angiography, which showed normal patent coronary arteries. This case report highlights a patient with a non-cardiac elevation of troponin, emphasizing the importance of considering non-cardiac causes when interpreting elevated troponin levels. The objective of this report is to raise awareness among healthcare professionals regarding the potential causes of false troponin elevation and promote a comprehensive diagnostic approach to minimize unnecessary interventions.

## Introduction

Elevated troponin levels are commonly associated with myocardial injury, particularly in the context of acute coronary syndrome. However, troponin elevations can also occur due to non-cardiac conditions, leading to diagnostic challenges. In this case report, we present a rare occurrence of a false elevation of troponin levels in a patient experiencing significant psychoemotional stress. Troponin is a cardiac biomarker commonly used to diagnose myocardial infarction (MI) and evaluate cardiac function. Elevated troponin levels are typically associated with cardiac injury or myocardial damage. However, in certain cases, troponin levels may be falsely elevated due to non-cardiac causes, including stress-induced myocardial stunning. Stress-induced myocardial stunning is a phenomenon characterized by reversible myocardial dysfunction in response to intense emotional or physical stress. It is commonly referred to as "broken heart syndrome" or takotsubo cardiomyopathy. This condition is often triggered by stressors such as the loss of a loved one, a traumatic event, or significant emotional distress. While the mechanisms underlying stress-induced myocardial stunning are not fully understood, it is believed that excessive catecholamine release and sympathetic hyperactivity play a significant role. These factors can lead to transient left ventricular dysfunction, resembling an acute coronary syndrome clinically and biochemically.

Here, we present a case of a patient who presented with elevated troponin levels in the absence of any significant cardiac pathology. Through a thorough evaluation and assessment of the patient's history, we identified significant psychoemotional stress as the likely cause of the false elevation of troponin levels. This case highlights the importance of considering non-cardiac causes for elevated troponin levels, particularly in patients with a history of significant psychological stress [1].

## Case presentation

A 63-year-old woman with cardiovascular risk factors, including hypertension and hyperlipidemia, presented to the emergency department (ED) with emotional stress associated with atypical chest pain. Part of her evaluation included troponins and EKG without ST changes. Serial troponins showed an initial value of 84, after which she was reassured and discharged.

**Figure 1:**
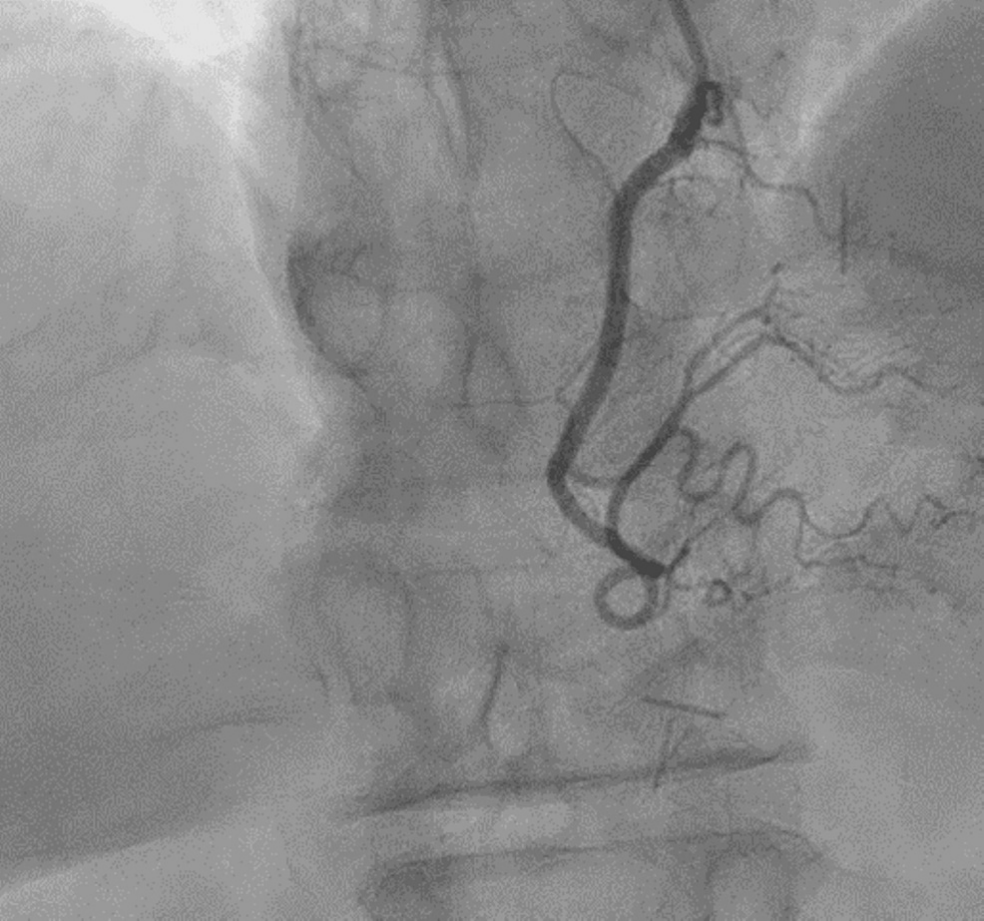
Coronary angiogram showing patent vessels.

The subsequent value came back 145, and she was contacted over the phone and advised to return for further evaluation. She was admitted overnight to the hospitalist service. A second EKG showed no changes compared to the initial EKG. An echocardiogram showed no wall motion abnormalities. Physical examination was normal. Heart sounds were rhythmic without murmurs, rubs, or gallops. The lungs were clear. There was no lower extremity edema and no elevation in the jugular venous pressure. Labs and imaging were reviewed. The assessment indicated that the patient has cardiovascular risk factors and significant elevation in troponin levels (a high delta between the first and third blood draws). We could not explain her elevated troponin to demand ischemia or other secondary causes. She did have elevated blood pressure on presentation, and she also had some complaints of intermittent palpitations, but there is no documentation of paroxysmal atrial arrhythmias or ventricular tachycardia. Her echocardiogram ruled out stress cardiomyopathy. For that reason, we recommended a coronary angiogram to definitively exclude coronary artery disease (CAD), spontaneous coronary artery dissection, or other coronary pathology, which revealed no evidence of significantly occlusive CAD. The patient's coronary angiogram shows the patent coronary blood vessels (Figure 1). 

## Discussion

Historically, troponins were implemented as the standard cardiac biomarkers in the 1990s [2]. A systematic search was conducted in academic databases, including PubMed and Google Scholar, using keywords such as "false positive troponin," "troponin assay accuracy," and "troponin diagnostic errors." Relevant studies and articles published between 2010 and 2023 were included. The inclusion criteria consisted of studies that focused on false positives in troponin assays, their causes, consequences, and potential solutions. The review identified several factors that contribute to false positive troponin results. These include non-ischemic causes of troponin elevation, such as renal dysfunction, pulmonary embolism, and sepsis. In addition, analytical factors, such as assay interference and analytical variability, can also lead to false positives. The consequences of false positive troponin results include unnecessary interventions, increased healthcare costs, and patient anxiety. Several strategies have been proposed to reduce false positives, including the use of high-sensitivity troponin assays, clinical algorithms, and serial troponin testing. The findings of this literature review highlight the multifactorial nature of false positive troponin results.

Overall, currently ongoing discussions are leaning more towards the fact that the delta change in troponin levels is more significant in establishing a diagnosis of myocardial injury than one isolated high number [3]. We usually take a couple of checks two hours apart. In our case, the troponin surged from 84 in the first sample to 145 in the second one, which signifies a decent delta change in a matter of two hours. This was actually the fundamental reason that encouraged us to take it seriously and proceed forward to coronary angiography.

However, other discussions also argue that despite some elevated troponin results might be associated with normal coronaries, it is very likely that those patients are still having non-coronary heart diseases. In this case, cardiac MRI is the recommended next step [3]. In our case, we scheduled a follow-up outpatient clinic visit in the Department of Cardiology in four to six weeks. If she continued to experience symptoms, an MRI might be considered in the future [4].

## Conclusions

False positive troponin results can have significant clinical implications, including unnecessary interventions and increased healthcare costs. Future research should focus on improving assay specificity, refining clinical algorithms, and evaluating the impact of different strategies on patient outcomes. By addressing false positives, clinicians can enhance the accuracy of acute myocardial infarction (AMI) diagnosis and improve patient care. It is essential for clinicians to consider the clinical context and potential confounding factors when interpreting troponin results. The use of high-sensitivity troponin assays has improved the accuracy of AMI diagnosis, but challenges still remain. Future research should focus on improving assay specificity, refining clinical algorithms, and evaluating the impact of different strategies on patient outcomes. By addressing false positives, clinicians can enhance the accuracy of AMI diagnosis and improve patient care.
